# Fine mapping and candidate gene analysis of proportion of four-seed pods by soybean CSSLs

**DOI:** 10.3389/fpls.2022.1104022

**Published:** 2023-01-18

**Authors:** Fubin Cao, Ruru Wei, Jianguo Xie, Lilong Hou, Chaorui Kang, Tianyu Zhao, Chengcheng Sun, Mingliang Yang, Ying Zhao, Candong Li, Nannan Wang, Xiaoxia Wu, Chunyan Liu, Hongwei Jiang, Qingshan Chen

**Affiliations:** ^1^ College of Agriculture, Key Laboratory of Soybean Biology in Chinese Ministry of Education, Northeast Agricultural University, Harbin, China; ^2^ Jilin Academy of Agricultural Sciences, Soybean Research Institute, Changchun, Jilin, China; ^3^ Jiamusi Branch Institute, Heilongjiang Academy of Agricultural Sciences, Jiamusi, Heilongjiang, China

**Keywords:** wild soybean, CSSLs, BSA, secondary segregation population, QTL for the proportion of four-seed pods, candidate gene

## Abstract

Soybean yield, as one of the most important and consistent breeding goals, can be greatly affected by the proportion of four-seed pods (PoFSP). In this study, QTL mapping was performed by PoFSP data and BLUE (Best Linear Unbiased Estimator) value of the chromosome segment substitution line population (CSSLs) constructed previously by the laboratory from 2016 to 2018, and phenotype-based bulked segregant analysis (BSA) was performed using the plant lines with PoFSP extreme phenotype. Totally, 5 ICIM QTLs were repeatedly detected, and 6 BSA QTLs were identified in CSSLs. For QTL (*qPoFSP13-1*) repeated in ICIM and BSA results, the secondary segregation populations were constructed for fine mapping and the interval was reduced to 100Kb. The mapping results showed that the QTL had an additive effect of gain from wild parents. A total of 14 genes were annotated in the delimited interval by fine mapping. Sequence analysis showed that all 14 genes had genetic variation in promoter region or CDS region. The qRT−PCR results showed that a total of 5 candidate genes were differentially expressed between the plant lines having antagonistic extreme phenotype (High PoFSP > 35.92%, low PoFSP< 17.56%). The results of haplotype analysis showed that all five genes had two or more major haplotypes in the resource population. Significant analysis of phenotypic differences between major haplotypes showed all five candidate genes had haplotype differences. And the genotypes of the major haplotypes with relatively high PoFSP of each gene were similar to those of wild soybean. The results of this study were of great significance to the study of candidate genes affecting soybean PoFSP, and provided a basis for the study of molecular marker-assisted selection (MAS) breeding and four-seed pods domestication.

## 1 Introduction

Soybean originated in China and then spread around the world. As an important food crop and oil crop, it has a cultivation history of more than 5000 years (Guo, 2004; Barik et al., 2018; Zhang et al., 2018). China is the world’s largest soybean consumer. In the past 10 years, China’s soybean consumption accounted for more than 30% of the world’s total, ranking first in the world (Liu and Fan, 2021). However, China’s soybean yield per unit is low, only 70% of the international average (Si and Han, 2021), and the domestic soybean supply is weak, with a supply rate of only approximately 15% (Liu and Fan, 2021). Improving soybean yield is an urgent problem to be solved in the world’s soybean industry.

The yield of soybean is affected by multiple factors, such as 100-seed weight, the number of pods per plant, the number of seeds per plant and the number of seeds per pod. These are all quantitative traits controlled by multiple genes and are easily affected by environmental factors (Xie et al., 2021). The total number of soybean pods per plant is one of the main factors limiting soybean yield. When the total number of pods varies little or the potential for increasing the number of pods is limited, PoFSP becomes the main limiting factor for the seed number per plant of soybean. Studies have shown that varieties with a high PoFSP have higher yield and productivity, and it is an effective way to increase the yield by increasing PoFSP of varieties, which has great practical value for increasing the yield. (Peng et al., 1994).

Compared with traditional breeding, the application of molecular marker technology has mostly overcome the difficulties introduced by environmental factors into phenotype identification (Song et al., 2012), and the emergence of MAS and QTL mapping has significantly shortened the breeding years (Hasan et al., 2021). MAS indirectly realizes the selection of QTL controlling a certain trait through the selection of genetic markers, so as to achieve the purpose of selecting the trait (Wakchaure et al., 2015). In 1988, Paterson mapped QTLs by using molecular markers in tomato for the first time (Paterson et al., 1988), and this method has been widely used in different crops (Song et al., 2005; Jiang et al., 2011; Yao et al., 2015; Botero-Ramírez et al., 2020). At present, a large number of studies have been carried out on QTL mapping of pod traits in soybean, and many QTLs related to four-seed pods have been mapped. Wang et al. mapped the QTL related to the number of pods and compared the heritability of one, two, three, and four seed pods by using soybean RILs (recombinant inbred line), and they found that the heritability of four-seed pods was higher and the main reason for the variation in seed pods number was the shedding of flower pods. In 2007, Wang et al. mapped two four-seed pods QTLs detected in two years by using CIM (composite interval mapping) method in soybean RILs (Wang et al., 2007). In 2008, Li et al. mapped three four-seed pods QTLs by using CIM method in soybean CSSLs (Li et al., 2008). In 2009, Zhou et al. mapped two four-seed pods QTLs by using CIM method in soybean RILs (Zhuo et al., 2009). In 2012, Gao et al. mapped 8 QTLs associated with four-seed pods by using CIM method in soybean RILs (Gao et al., 2012). In 2013, Yang et al. mapped 4 four-seed pods QTLs by using the multi-environment joint analysis method in soybean RILs (Yang et al., 2013b). In 2018, Ning et al. mapped 36 and 12 four-seed pods QTLs in soybean by using single marker analysis, CIM and multiple interval mapping methods RIL6013 and RIL3613, respectively (Ning et al., 2018). In 2021, Li et al. constructed 3 RHL s and used single marker analysis to fine-map a four-seed pods QTL, narrowing the QTL interval to 321 kb (Li et al., 2021).

In 1991, BSA method was first proposed by Michelmore et al. (Michelmore et al., 1991). Its core purpose is to use extreme material of the target trait in the population to construct two mixed pools and to locate the target gene by analyzing the degree of association between the polymorphic molecular markers and the phenotype (Zou et al., 2016). Compared to using near-isogenic lines (NILs) to identify markers in specific regions of the genome, BSA is faster and less labor. And BSA significantly reduces the cost of sequencing and analysis because only a few extreme individuals in a population are needed (Yang et al., 2013a).

The quality of the genetic population directly affects the effect, difficulty and application scope of the constructed genetic map s. If the genome differences between the parents are greater, the effect of exchange and recombination between their chromosomes will be more obvious, and the DNA polymorphism and phenotypic variation will be more abundant (Xi et al., 2005). Cultivated soybean was domesticated from annual wild soybean (Broich and Palmer, 1980), and there are significant differences between them. In this study, CSSLs were constructed using cultivated soybeans and wild soybeans. There are abundant variations in this population, and the ability to detect QTLs is strong. Although the genetic variation information is rich, the diversity of their genetic background will affect the accuracy of QTL mapping by using traditional mapping population such as F_2_, BC, DH, RIL and others (Zhao et al., 2009; Qiao et al., 2016). Compared with the primary mapping population, the genetic background of CSSLs is purer, which not only eliminates genetic noise but also improves the accuracy of QTL mapping (Li, 2014). At present, CSSLs have been applied to 20 crops, and researchers have used these CSSLs to map a large number of QTLs (He et al., 2014; Li et al., 2016; Alyr et al., 2020; Yuan et al., 2020; Xu et al., 2022).

In this study, CSSLs constructed previously in the laboratory was used as the material. Combined with the results of ICIM method and BSA method in CSSLs, PoFSP QTL in soybean was initially mapped, and the secondary segregated populations were constructed for fine mapping. The genes were screened out in the fine mapping interval, and the candidate genes for PoFSP were analyzed and predicted by expression and haplotype analysis.

## 2 Materials and methods

### 2.1 Plant material and field management

The population orientation material for this study was CSSLs containing 208 lines constructed from SN14 (the recurrent parent) and ZYD00006 (Jiang et al., 2020), which were planted in Xiangyang Farm, Harbin, China, from 2016 to 2018 (45°45′N, 126°38′E). A completely random block design was adopted, and each line was planted in one row, repeated three times. The row length was 5 m, the row spacing was 65 cm, and the plant spacing in each single-row plot was 6 cm, with approximately 80 plants. Fine mapping population was constructed by backcrossing R92 with extremely high PoFSP in CSSLs with recurrent parent SN14. The R92-F_1_ seeds obtained in the same year were propagated in Nannan, Hainan, and the R92-F_2_ was planted in Xiangyang Farm the following year, and 121 R92-F_2_ individuals were obtained. RHLs(H1) was constructed from an individual in R92-F_2_ whose genotype was completely heterozygous in the target interval and whose remaining background was relatively pure. The resource population was also planted on the Sunshine Farm, and the planting method was the same as that of CSSLs. The field management of the above materials followed general agricultural practices.

### 2.2 Phenotypic identification and statistical analysis

For CSSLs, 5 complete plants were selected for each material at the mature stage in the field to test the number of pods and the number of seeds per pod at the end of September every year before harvesting. Each plant of R92-F_2_ and H1 s was also investigated in the field before harvesting for the number of pods and the number of seeds per pod, and PoFSP was calculated. The measurement standard referred to the “Soybean Germplasm Description Specification and Data Standard” (Zhou et al., 2015).

The phenotypic data were sorted using Microsoft^®^ Excel 2016, the descriptive analysis was performed using SPSS 17.0, and the significance analysis was performed using one-way ANOVA. BLUE was calculated by the BLUE calculation option in AOV module of ICIMapping 4.2 software. The parameters used were each line of CSSLs as a fixed factor and the years and repetition as random factors.

### 2.3 Bulk segregant analysis

In this study, phenotype-based BSA was performed by 30 materials with high PoFSP (PoFSP > 17.14%) and 30 materials with low PoFSP (PoFSP< 1.46%) in 208 CSSLs. A total of 3,716,818 SNPs were detected, and 3,105,246 high-quality SNPs were obtained after screening out. The Euclidean Distance method was used to analyze the association between the markers and PoFSP in the sequencing results:

ED=


$$\sqrt{{\left(\text{A}1-\text{A}2\right)}^{2}+{\left(\text{C}1-\text{C}2\right)}^{2}+{\left(\text{G}1-\text{G}2\right)}^{2}+{\left(\text{T}1-\text{T}2\right)}^{2}}$$

A1 and A2 was in the same position. A1 represented the occurrence frequency of A in the high phenotype pool, and A2 represented the occurrence frequency of A in the low phenotype pool. C, G, and T were the same as A. The mean ± 3 times the standard deviation (0.02238) was taken as the threshold value to judge whether the marker and the trait were closely linked, and if it exceeded the specified threshold, it was considered to be related to the trait.

### 2.4 QTL mapping for PoFSP

The whole genome resequencing data of CSSLs (Zheng et al., 2022) constructed in the laboratory and PoFSP data and BLUE values from 2016-2018 were used for QTL mapping. QTL mapping employed the RSTEP-LRT-ADD model with the 1,000 permutations calculation of ICIMapping 4.2 software. Parameters setting was “By condition number” = −1,000 (equivalent to deleting duplicate markers), “PIN” = 0.001 (PIN: the largest-value for entering variables in stepwise regression of residual phenotype on marker variables). DNA from fresh leaves was extracted using the cetyltrimethylammonium ammonium bromide (CTAB) plant tissue DNA extraction method, and the DNA concentration and purity were determined using a NanoDrop 2000C (Sunnyvale, California, USA) ultradifferential photometer and 1.5% agarose gel electrophoresis. The SSR markers were used as molecular markers. After the polymerase chain reaction (PCR) of Panaud, the genotype of the secondary segregated population was detected by 8% PAGE separation (Doyle, 1990). The genetic maps of the secondary segregating populations were constructed by the MAP module in ICIMapping 4.2 software. QTL mapping employed ICIM-ADD model with 1,000 permutations calculation of ICIMapping 4.2 software. And the threshold of ICIMapping for QTL detection was chose p ≤ 0.05.

### 2.5 qRT−PCR analysis of candidate genes

According to different stages of flower bud differentiation, the flower tissues of soybean were sampled three days before flowering, two days before, one day before, on the day of flowering, three days after flowering and five days after flowering and stored in liquid nitrogen. The sampling materials were selected from the R92-F_2_. L-9 and L-14 which had the genotype of ZYD00006 had a high PoFSP (PoFSP > 35.92%). L-72 and L-93 which had the genotype of SN14 had a low PoFSP (PoFSP< 17.56%). Three biological replicates were sampled per material. After grinding the samples in liquid nitrogen, RNA from the plant tissues was extracted using the TRIzol method. The RNA concentration and purity were determined using a NanoDrop 2000C (Sunnyvale, California, USA) ultradifferential photometer and 1.5% agarose gel electrophoresis. Reverse transcription of the extracted RNA into cDNA using the Tianhe Real-time quantitative PCR (RT−qPCR) kit was performed using SYBR qPCR Mix (Vazyme, Q711, Vazyme Biotech, Nanjing, China) on the Light Cycler 480 System (Roche, Roche Diagnostics, Basel, Switzerland). qRT−PCR primer sequences for the candidate genes were designed using Premier 5.0. Using *GmACTIN* as an internal reference, the average of three biological replicates was taken, and the relative expression levels of the candidate genes were calculated by the 2^-ΔCt^ method.

### 2.6 Haplotype analysis

Haplotype analysis of PoFSP candidate genes was conducted using 527 soybean germplasm resources. Combined with the sequence information of the promoter region and CDS region of the candidate gene in the reference genome, local sequence alignment was performed on the resequencing data of the candidate gene between the parents of CSSLs to find the difference sites and analyze the variation in the promoter elements and CDS region translation products. The haplotype distribution of the candidate genes in the resource population was counted by DnaSP 5.0 software. The major haplotypes were divided by the number of varieties exceeding 5% of in resource population, and Haploview 4.2 software was used to perform linkage disequilibrium (LD) analysis on the mutation sites among the major haplotypes. Using one-way AOV(analysis of variance) in IBM SPSS Statistics 22 software, combined with the phenotypic data of PoFSP in the resource population in 2021, the significance of the difference between the phenotypes of each major haplotype material was compared.

## 3 Results

### 3.1 QTL analysis in CSSLs

**Figure 1:**
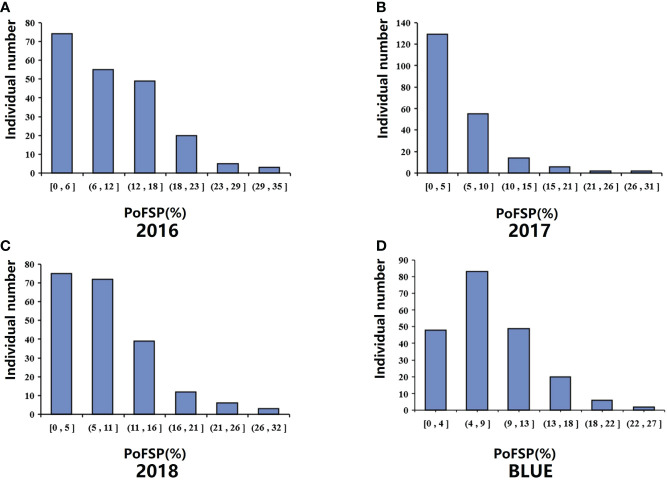
Phenotypic identification of PoFSP in CSSLs. **(A)**: PoFSP of CSSLs in 2016. **(B)**: PoFSP of CSSLs in 2017. **(C)**: PoFSP of CSSLs in 2018. **(D)**: PoFSP of CSSLs in BLUE.

**Figure 2:**
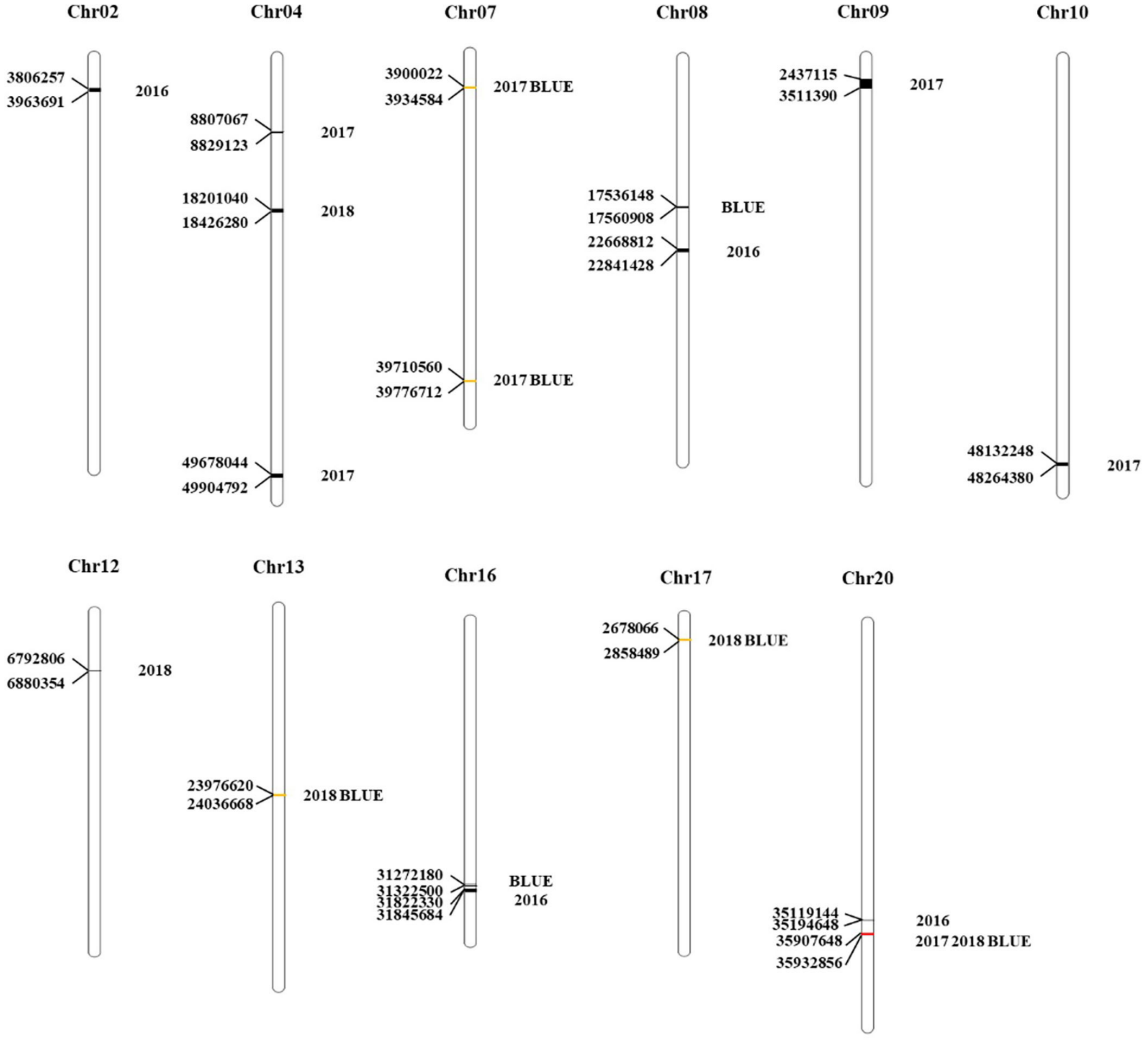
Distribution of PoFSP QTLs on chromosomes in CSSLs. The unit of each position in the figure is bp.

QTL analysis was performed based on the phenotypic data (Zheng et al., 2022) and BLUE value data of PoFSP in CSSLs from 2016 to 2018 (**Figure 1**). A total of 17 QTLs were detected, distributed on 13 chromosomes (**Supplementary Table 1**), of which 5 were repeatedly detected in two or more years. *qPoFSP07-1* and *qPoFSP07-2* on Chr07 were detected in 2017 and BLUE, with maximum PVE (phenotypic variance explained) of 3.87% and 8.76%, respectively; *qPoFSP13-1* on Chr13 was detected in 2018 and BLUE, with a maximum PVE of 9.18%; *qPoFSP17-1* on Chr17 was detected in 2018 and BLUE, with a maximum PVE of 5.14 %; *qPoFSP20-2* on Chr20 was detected in 2017, 2018 and BLUE, with a maximum PVE of 30.69 % (**Table 1**; **Figure 2**). The above 5 QTLs were repeatedly detected in two or more years, which was more reliable than the QTLs that appeared in a single year. And the five QTLs would be the focus of follow-up research.

**Table 1:** PoFSP QTLs detected in CSSLs for two or more years.

Years	Name	Chromosome	LOD	PVE (%)	ADD	Start (bp)	End (bp)
2017	*qPoFSP07-1*	Chr07	3.46	3.87	0.03	3900022	3934584
2017	*qPoFSP07-2*	Chr07	7.19	8.76	0.02	39710561	39776712
2017	*qPoFSP20-2*	Chr20	6.94	8.35	-0.02	35907647	35932855
2018	*qPoFSP13-1*	Chr13	4.22	6.69	0.02	23976621	24036668
2018	*qPoFSP17-1*	Chr17	3.32	5.14	-0.03	2678066	2858489
2018	*qPoFSP20-2*	Chr20	9.13	15.12	-0.04	35907647	35932855
BLUE	*qPoFSP07-1*	Chr07	2.96	1.94	0.02	3900022	3934584
BLUE	*qPoFSP07-2*	Chr07	10.50	7.44	0.02	39710561	39776712
BLUE	*qPoFSP13-1*	Chr13	12.63	9.18	0.02	23976621	24036668
BLUE	*qPoFSP17-1*	Chr17	4.65	3.05	-0.02	2678066	2858489
BLUE	*qPoFSP20-2*	Chr20	33.31	30.69	-0.05	35907647	35932855

the “+” additive indicates that the additive effect comes from the allele of the wild parent ZYD00006.

### 3.2 Analysis of the BSA sequencing results

**Figure 3:**
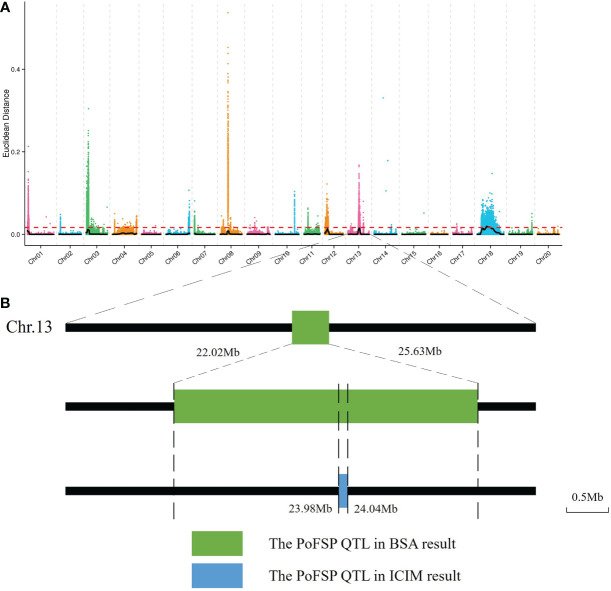
BSA results. **(A)**: The distribution of Euclidean distance (ED)-associated values on chromosomes. **(B)**: Preliminary mapping process of QTLs for PoFSP. ^a^: The abscissa is the chromosome name. The color points represent the ED value of each single nucleotide polymorphism (SNP) locus. The black line is the fitted ED value, and the red dotted line represents the significantly associated threshold. The higher the ED value is, the better the correlation.

BSA-seq was performed on the extreme materials of PoFSP in CSSLs. A total of 6 PoFSP QTLs were detected, which were distributed on Chr01, Chr03, Chr08, Chr12, Chr13 and Chr18, and the interval size was 0.09 Mb, 3.67 Mb, 0.10 Mb, 2.57 Mb, 3.61 Mb, and 27.4 Mb, respectively (**Supplementary Table 2**; **Figure 3A**). The QTL located on chromosome 13 coincides with the *qPoFSP13-1* in CSSLs mapping results, with a size of 3.61 Mb (**Figure 3B**). *qPoFSP13-1* was repeated twice in ICIM results and overlapped with BSA result s, showing a strong association with PoFSP phenotype. Therefore, *qPoFSP13-1* was further fine-mapped as an important candidate QTL for PoFSP.

### 3.3 Construction and fine mapping of secondary separation populations

**Figure 4:**
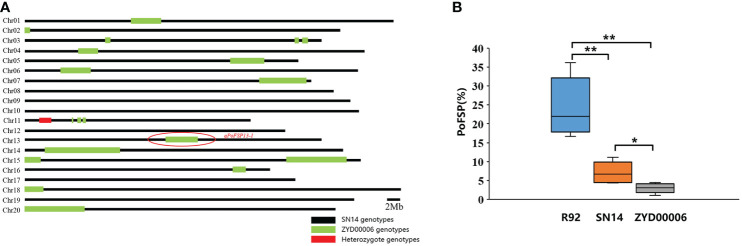
Genotypic background and phenotypic identification of R92. **(A)**: The distribution of the ZYD00006 segment in the R92 genome. **(B)**: PoFSP in R92 and the parents. ^b^: “*” means the significant difference at the 0.05 level, and “**” means the significant difference at the 0.01 level.

According to the location information of the candidate QTL (*qPoFSP13-1*) on the chromosome and the whole genome resequencing information of CSSLs, combined with the introduction of the ZYD00006 segment in the whole genome, a total of 16 lines from 208 CSSLs were screened out for the ZYD00006 homozygous genotype introduction segment completely covered the candidate QTL on chromosome 13. The results of phenotypic identification showed that PoFSP in Line R92 was significantly higher than that of its parents and reached an extremely significant level (P ≤ 0.01), which was a superparental material, and its target introduction segment size was 4.9 Mb (**Figures 4A, B**). Therefore, R92 was selected as the follow-up research material.

**Figure 5:**
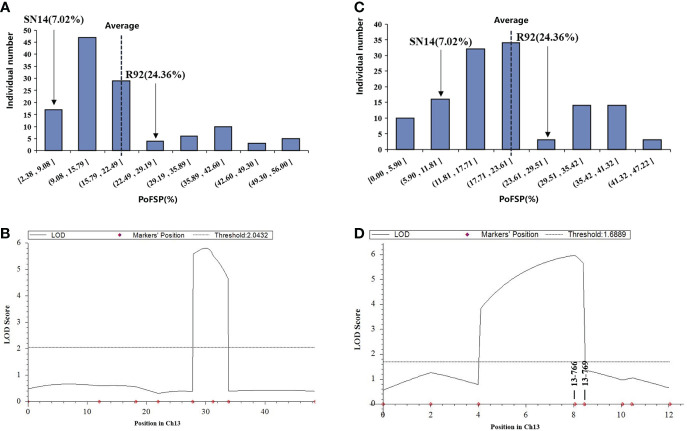
Phenotypic identification and QTL results of fine-mapping populations. **(A)**: Frequency histogram of the phenotypic distribution of PoFSP in the R92-F_2_. **(B)**: QTL mapping results of ICIM method in R92-F_2_. **(C)**: Frequency histogram of the phenotypic distribution of PoFSP in the H1. **(D)**: QTL mapping results of ICIM method in H1.

The secondary segregation population R92-F_2,_ including 121 plants, was constructed by crossing R92 with the recurrent parent. PoFSP was calculated for the R92-F_2_. The phenotypic data showed that the highest and lowest PoFSP in R92-F_2_ were 56.00% and 2.38%, respectively. The R92-F_2_ was rich in phenotypic variation with obvious differences and was suitable for QTL analysis (**Figure 5A**; **Table 2**). Eight pairs of SSR markers evenly distributed on the R92 target introduction segment and polymorphic between parents were screened out, linkage analysis was performed on the R92-F_2_, and the QTL interval was located between markers 13-755 and 13-769. The length of the candidate interval was 1.2 Mb, and PVE was 11.06%. The ADD results showed that the additive effect of the gain came from ZYD00006 (**Figure 5B**; **Table 3**).

**Table 2:** Phenotypic statistics of PoFSP in the secondary segregated population.

Population	Number	Average PoFSPN (%)	Minimum (%)	Maximum (%)		PoFSPN for SN14 (%)	PoFSPN for R92 (%)
R92-F_2_	121	19.16	2.38	56.00	7.02		24.36
H1	126	20.22	0.00	47.22			

**Table 3:** Mapping of PoFSP QTLs by secondary segregated population ICIM.

Population	Chromosome	Left marker	Right marker	LOD	PVE (%)	Add	Size (Mb)
R92-F_2_	Chr13	13-755	13-769	2.78	11.06	0.06	1.2
H1	Chr13	13-766	13-769	6.00	19.47	0.05	0.1

the “+” additive indicates that the additive effect comes from the allele of the wild parent ZYD00006.

**Figure 6:**
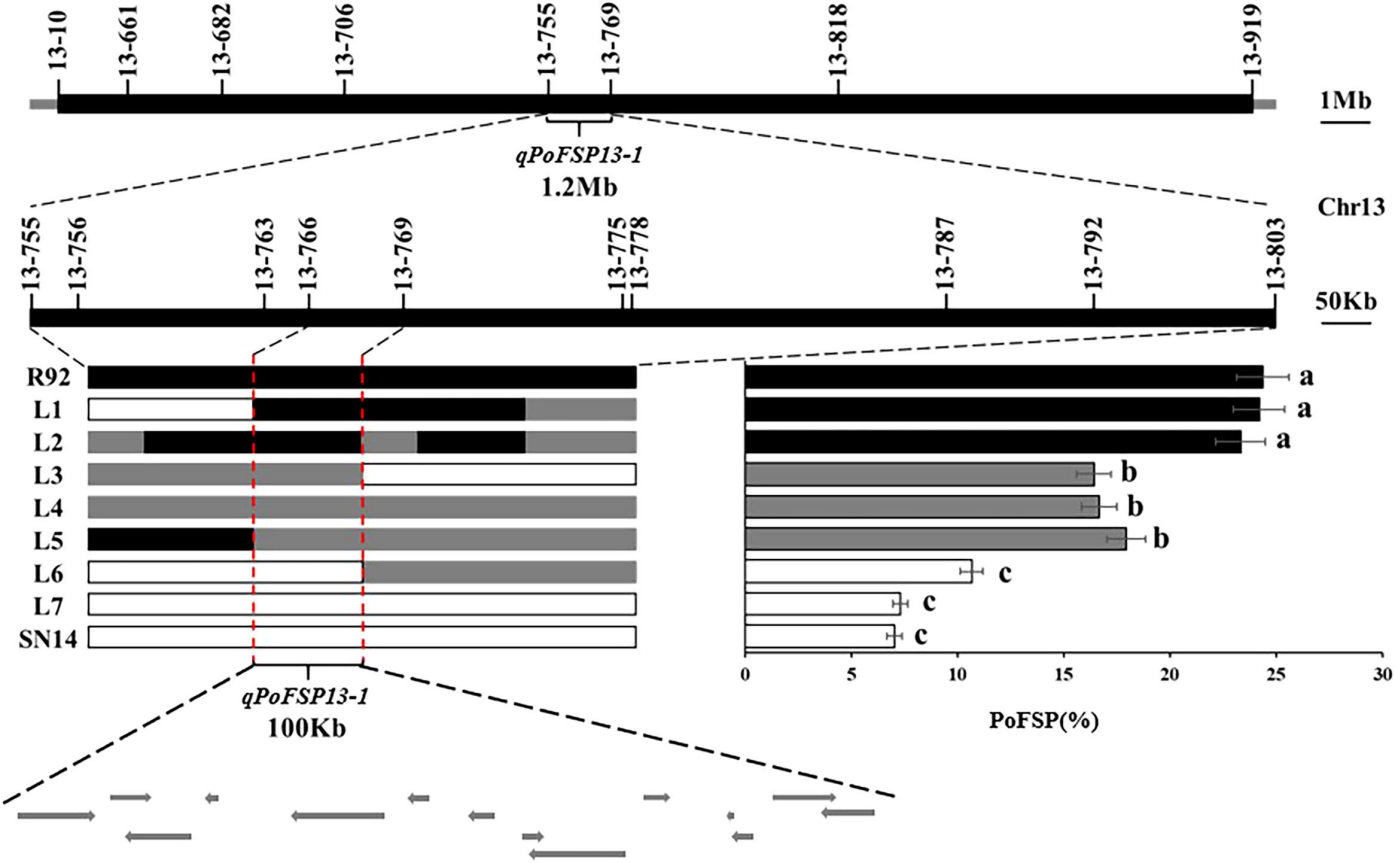
Schematic diagram fine mapping of *qPoFSP13-1.* 
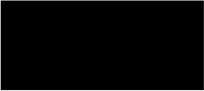
represents the ZYD00006 genotype, 
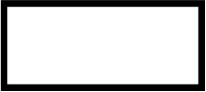
represents the SN14 genotype, and 
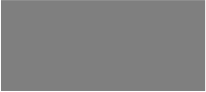
represents the heterozygous genotype. R92, L1 and L2 were the same genotypes as ZYD00006; L3, L4 and L5 were heterozygous genotypes of ZYD00006, and L6 and L7 were the same genotypes as SN14. Different letters indicate the significant differences, while the same letters indicate the non-significant differences.

RHLs(H1) is constructed from an individual in R92-F2 whose genotype is completely heterozygous in the target interval and whose remaining background is relatively pure. In H1 containing 126 plants, the highest PoFSP was 47.22%, and the lowest PoFSP was 0.00. The H1 was rich in phenotypic variation with obvious differences and was suitable for QTL analysis (**Figure 5C**; **Table 2**). Ten pairs of SSR markers evenly distributed in the initial mapping QTL interval and polymorphic between parents were screened out, and H1 was genotyped. A total of one PoFSP QTL with PVE of 19.47% was detected. The ADD results showed that the additive effect of gain still came from ZYD00006 (**Figure 5D**; **Table 3**). Finally, *qPoFSP13-1* was fine-mapped between the two flanking markers 13-766 and 13-769, and the candidate interval length was 100 kb (**Figure 6**).

### 3.4 Gene screening and prediction within the QTL interval

**Figure 7:**
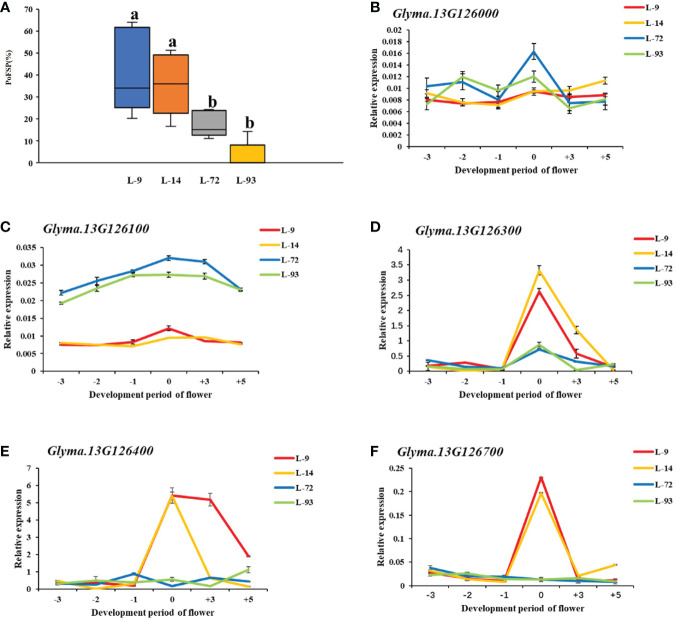
Expression analysis of candidate genes. **(A)**: Phenotypic identification of extreme materials. **(B–F)**: Real-time PCR results of the candidate genes. ^a^: Different letters indicate the significant differences at the 0.05 level, while the same letters indicate the nonsignificant differences. ^b-f^: -3, -2, -1, 0, + 3, +5 represent 3 days before flowering, 2 days before flowering, 1 day before flowering, the day of flowering, 3 days after flowering, 5 days after flowering, respectively.

According to the Williams 82.a2.v1 reference genome sequence information in SoyBase(https://www.soybase.org/) and Phytozome(https://phytozome-next.jgi.doe.gov/), a total of 14 PoFSP candidate genes were annotated in *qPoFSP13-1* (**Supplementary Table 3**). Combined with the resequencing data of the parents of CSSLs, the sequence analysis results showed 13 candidate genes had InDel mutations and 14 candidate genes had SNP mutations in the promoter region, 8 candidate genes had nonsynonymous mutations and 2 candidate genes had InDel mutations in the coding region (**Supplementary Table 4**; **Supplementary Table 5**; **Supplementary Table 6**). The expression levels of 14 candidate genes were analyzed in 6 periods before and after flowering. qRT-PCR results showed that *Glyma.13G126000* was expressed at low levels before flowering, on the day of flowering and after flowering in the high-extreme materials L-9 and L-14. However, its expression level gradually increased before flowering, peaked on the day of flowering, and then gradually decreased in the low-extreme materials L-72 and L-93. *Glyma.13G126100* was also expressed at low levels during each period of in the high-extreme materials L-9 and L-14. However, its expression in low-extreme materials L-72 and L-93 was higher than that in high-extreme materials as a whole, and its expression also increased gradually before flowering and peaked on the day of flowering, and then gradually decreased in L-72 and L-93. The expression levels of *Glyma.13G126300, Glyma.13G126400* and *Glyma.13G126700* gradually increased before flowering, peaked on the day of flowering, and then gradually decreased in the high-extreme materials L-9 and L-14. However, it showed low expression in each period in the low-extreme materials L-72 and L-93 (**Figure 7**). According to the above results, it is predicted that *Glyma.13G126000* and *Glyma.13G126100* might be negative regulators of PoFSP, while *Glyma.13G126300*, *Glyma.13G126400* and *Glyma.13G126700* might be positive regulators of PoFSP. In conclusion, *Glyma.13G126000*, *Glyma.13G126100*, *Glyma.13G126300*, *Glyma.13G126400*, and *Glyma.13G126700* were screened out as candidate genes affecting PoFSP in soybean.

### 3.5 Candidate gene haplotype analysis

Using DnaSP 5.0 software, haplotype analysis was performed on the above five candidate genes for PoFSP in the resource population contained 527 varieties. AOV was used to analyze the significance of the phenotypic differences among the major haplotypes. The major haplotypes were divided according to the standard that the number of varieties exceeded 5% of the resource population. All five candidate genes had two or more major haplotypes in the resource population.

**Figure 8:**
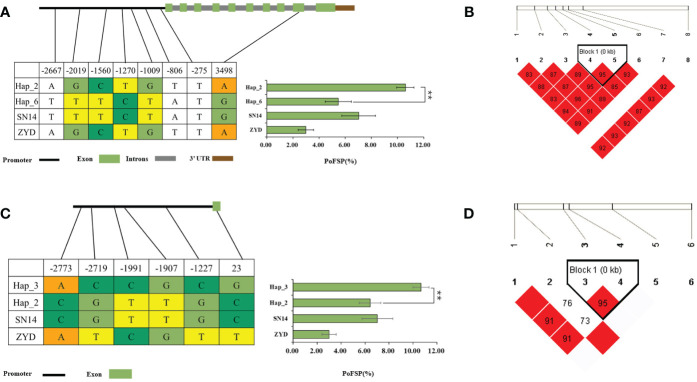
Haplotype analysis results of *Glyma.13G126000* and *Glyma.13G126100*.**(A)**: Correlation analysis of the major haplotype phenotype in *Glyma.13G126000.* **(B)**: LD analysis of SNPs among major haplotypes of*Glyma.13G126000*. **(C)**: Correlation analysis of the major haplotype phenotype in *Glyma.13G126100.* **(D)**: LD analysis of SNPs among major haplotypes of*Glyma.13G126100.* ^a,c^: “**” indicates a significant difference at the 0.01 level.

*Glyma.13G126000* had 2 major haplotypes in the resource population, Hap_2 had 367 resource varieties, and Hap_6 has 84 resource varieties. The results of AOV showed that there was an extremely significant difference in PoFSP between Hap_2 and Hap_6. Through SNP analysis, *Glyma.13G126000* had a total of 8 differential SNPs in the resource population, of which 7 SNPs were between Hap_2 and Hap_6. SNP-1009 was located on a motif sequence in the promoter region of *Glyma.13G126000*, and its mutation might affect the binding of transcription factors to promoters. SNP-1270 mutation did not cause changes in promoter elements, but it was tightly linked to SNP-1009 according to the LD analysis. SNP-2667, SNP-2019, SNP-1560 and SNP-806 were also located in the promoter region, but none of their mutations caused changes in promoter elements. In the coding region, SNP3498 was A in Hap_2, which was consistent with ZYD00006, while it was G in Hap_6, which was consistent with SN14 (**Figures 8A, B**). This site mutation caused the corresponding translation product to change from Glu to Arg, which might affect the changes in gene structure or function.

*Glyma.13G126100* had a total of 2 major haplotypes in the resource population, Hap_2 had 114 resource varieties, and Hap_3 had 362 resource varieties. The results of AOV showed that there was an extremely significant difference in PoFSP between Hap_2 and Hap_3. Through SNP analysis, *Glyma.13G126100* had a total of 6 differential SNPs in the resource population. SNP-1991 was located on the promoter of *Glyma.13G126100*, and its mutation resulted in an alteration of an MYB transcription factor-related element that might affect gene transcription. SNP-1907 did not cause changes in promoter elements, but it was tightly linked to SNP-1991 by LD analysis. SNP-2773, SNP-2719, and SNP-1227 were also located in the promoter region, but none of their mutations caused changes in the promoter elements. In the coding region, SNP23 was C in Hap_2, which was consistent with SN14 and encoded Pro, while it was G in Hap_3, which was a dominant mutation type different from its parents and encoded Gly (**Figures 8C, D**). Variations in the translation products caused by the mutations at this site might affect the gene structure or function.

**Figure 9:**
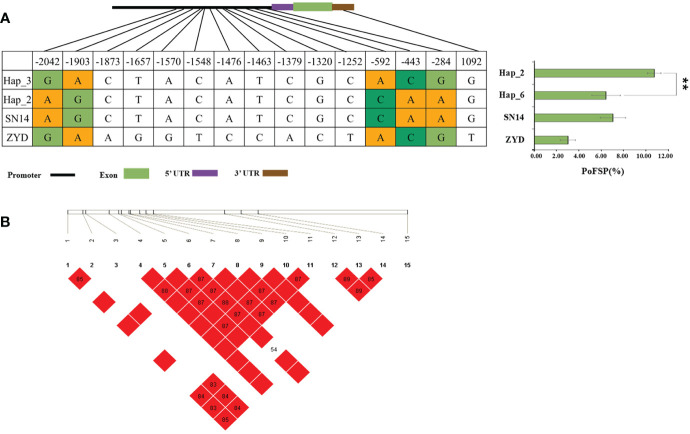
Haplotype analysis results of *Glyma.13G126300*. **(A)**: Correlation analysis of the major haplotype phenotype in *Glyma.13G126300*. **(B)**: LD analysis of SNPs among major haplotypes of*Glyma.13G126300*. ^a^: “**” indicates a significant difference at the 0.01 level.

*Glyma.13G126300* had a total of 2 major haplotypes in the resource population, Hap_2 had 78 resource varieties, and Hap_3 had 364 resource varieties. The results of AOV showed that there was an extremely significant difference in PoFSP between Hap_2 and Hap_3. Through SNP analysis, *Glyma.13G126300* had a total of 15 differential SNPs in the resource population, of which 5 SNPs were between Hap_2 and Hap_3, and all of them were located in the promoter region. There was a motif sequence on SNP-443 and SNP-284 respectively, and thier mutations caused changes in the motif sequence, which might affect transcription factor binding. According to LD analysis, SNP-592 was closely linked with SNP-443 and SNP-284, but SNP-592 did not cause the promoter element to change (**Figures 9A, B**).

**Figure 10:**
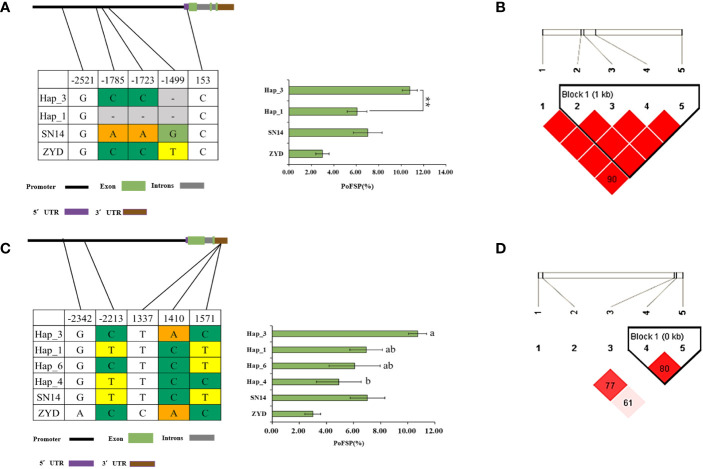
Haplotype analysis results of *Glyma.13G126400* and *Glyma.13G126700*. **(A)**: Correlation analysis of the major haplotype phenotype in *Glyma.13G126400*. **(B)**: LD analysis of SNPs among major haplotypes of*Glyma.13G126400*. **(C)**: Correlation analysis of the major haplotype phenotype in *Glyma.13G126700*. **(D)**: LD analysis of SNPs among major haplotypes of*Glyma.13G126700.* ^a,c^: “**” indicates a significant difference at the 0.01 level.

*Glyma.13G126400* has a total of 2 major haplotypes in the resource population, Hap_1 has 128 resource varieties, and Hap_3 has 364 resource varieties. The results of AOV showed that there was an extremely significant difference in PoFSP between Hap_1 and Hap_3. Through SNP analysis, *Glyma.13G126400* has 5 differential SNPs in the resource population, of which 3 SNPs were between Hap_1 and Hap_3, and all of them are located in the promoter region. According to LD analysis, SNP-1785 and SNP-1499 are closely linked with SNP-1723, but mutation of them will not cause changes in the promoter elements, and there is a TATA-box sequence at SNP-1723. Mutations in this sequence may affect the production of TATA-box-binding proteins, possibly resulting in the inhibition of RNA polymerase transcription factor synthesis (**Figures 10A, B**).

*Glyma.13G126700* had a total of 4 major haplotypes in the resource population. Hap_1 had 75 resource varieties, Hap_3 had 361 resource varieties, Hap_4 had 28 resource varieties, and Hap_6 had 28 resource varieties. AOV and multiple comparisons showed that only Hap_3 and Hap_4 had a significant difference in PoFSP. Through SNP analysis, *Glyma.13G126700* had a total of 5 SNPs in the resource population, of which 2 SNPs were between Hap_3 and Hap_4. SNP-2213 was located in a CAAT-box sequence on the promoter, and its mutation might lead to changes in the CAAT-box sequence, which might affect the normal initiation of gene transcription. SNP1410 was located in the 3’UTR, and its mutation might affect the stability of the gene structure. According to LD analysis, although SNP1571 had no mutation in Hap_3 and Hap_4, it was closely linked with SNP1410. In Hap_4, the linkage between the two was broken, which might also destroy the 3’UTR structure and affect the stability of the gene structure (**Figures 10C, D**).

## 4 Discussion

Soybean originated in China and is an important oil and cash crop (He et al., 2022). The yield of soybean is affected by a variety of yield factors, such as the number of pods per plant, the number of seeds per pod, 100-seed weight and the number of seeds per plant. It is a quantitative trait susceptible to environmental factors and controlled by multiple genes (Sun et al., 2012). As one of the important factors affecting yield, it is of great significance to improve PoFSP for yield research.

According to the data published on the SoyBase, 425 QTLs related to soybean four-seed pods have been reported to date. In this study, 12 QTLs detected in a single year and 5 QTLs repeatedly detected in two years or more were mapped by PoFSP data and BLUE value in CSSLs from 2016 to 2018. Compared with previous research results, *qPoFSP20-2* detected in this study in 2017, 2018 and BLUE value highly overlapped with the *Ln* loci mapped by Fang et al. (Fang et al., 2013) in 2013, reflecting the reliability of CSSLs mapping results. *qPoFSP20-2* was detected three times and its LOD and PVE were the most prominent. But *qPoFSP13-1* was detected two times and its LOD and PVE were also outstanding, and coincided with BSA results. In this study, the latter QTL was selected as the research object more convincingly.

In previous studies, the *Ln* gene named after a narrow leaflet has been frequently studied, and it was an important gene affecting the number of four-seed pods (Takahashi, 1934; Domingo, 1945; Peng et al., 1994). A large number of studies have proven that soybean leaf type and four-seed pods were controlled by pleiotropic genes (Johnson and Bernard, 1962; Weiss, 1970; Dinkins et al., 2002). Fang et al. successfully cloned the *Ln* gene and found through functional analysis that the *Ln* gene could increase the number of four-seed pods (Fang et al., 2013). Moreover, while the *Ln* gene has been studied in depth, other genes related to PoFSP have also been discovered one after another. Wang et al. successfully cloned the soybean *GmCYP78A10* gene in 2015 and found that *GmCYP78A10* can increase PoFSP (Wang et al., 2015). Wang et al. analyzed transcriptome sequencing to obtain differentially expressed genes in 2022 and found that the *Glyma.19G240800* gene was homologous to the Arabidopsis cytochrome P450 gene *Klu* (Wang et al., 2022). Poretska et al. found that the *Klu* gene was required for female meiosis during ovule development (Poretska et al., 2020). In this study, CSSLs and constructed RHLs were used to fine-map a QTL for PoFSP (*qPoFSP13-1*). In addition, *qPoFSP13-1* has not been reported in previous studies, and no previously reported PoFSP genes were found in *qPoFSP13-1*, including the genes mentioned above. Therefore, this study might predict a new gene affecting PoFSP in soybean.

China’s wild soybean resources are rich and widely distributed. Compared with cultivated soybeans, wild soybeans have more pods and a higher protein content (Jin et al., 2017). The application of wild soybean varieties can effectively solve the problem of genetic simplification in the breeding of cultivated soybean. This study used PoFSP phenotype data and BLUE values from 2016-2018 of the wild soybean CSSLs and BSA results to comap the QTL(*qPoFSP13-1*), and the genes identified by fine mapping with high reliability. The mapping results showed that the QTL had an additive effect of gain from the wild parent. Moreover, the genotypes of the five candidate genes in the resource population with higher haplotypes of PoFSP were similar to those of wild soybean. This indicated that the original gene haplotype affecting PoFSP in wild soybean has been preserved during the process of soybean domestication. These results are of great significance for the study of four-seed pods inheritance.

In this study, five candidate genes (*Glyma.13G126000, Glyma.13G126100, Glyma.13G126300, Glyma.13G126400, and Glyma.13G126700*) were analyzed and predicted in the fine-mapping interval. All these genes mutated between cultivated and wild parents. According to the Williams 82 reference genome gene annotation information published by the SoyBase and Phytozome websites, *Glyma.13G126000*, *Glyma.13G126100* and *Glyma.13G126300* had no annotated gene functions. This study preliminarily predicted that *Glyma.13G126000* and *Glyma.13G126100* might be negative regulators of PoFSP in soybean through the analysis of gene expression in plant lines having antagonistic extreme phenotype, but *Glyma.13G126300* might be a positive regulator of PoFSP in soybean. Additional research is needed to verify its regulatory mechanism on soybean four-seed pods. *Glyma.13G126400* belongs to the L14b domain of ribosomal proteins. Ribosomal proteins are an important part of the ribosome and have an important impact on the reproductive process of cells (Jin et al., 2018). Wu et al. (2012) found that the mitochondrial ribosomal protein GCD1 was required for the maturation of Arabidopsis female gametes (Wu et al., 2012). In 2014, Agustin et al. found that the Arabidopsis ribosomal protein L27a promoted female gametophyte development in a dose-dependent manner (Zsögön et al., 2014). Zhang et al. found in 2015 that the Arabidopsis mitochondrial ribosomal protein gene *HEART STOPPER* was required for seed development (Zhang et al., 2015). Yan et al. found in 2016 that the Arabidopsis ribosomal protein L18aB was necessary for male gametophyte and embryonic development, and Xie et al. found in 2018 that L18aB was involved in the formation of embryonic stalks in early embryonic development, further confirming that L18aB was required for reproductive development (Yan et al., 2016; Xie et al., 2018). In 2017, Lu et al. found that the Arabidopsis mitochondrial ribosomal protein S9 M was required for central cell maturation and endosperm development, and in 2020, they found that S9 M was also involved in male gametogenesis and seed development (Lu et al., 2017; Lu et al., 2020). In 2020, Xiong et al. found that the Arabidopsis ribosomal protein L27a participated in the formation of gametophytes by interacting with its molecular chaperone KETCH1 (Xiong et al., 2020). Luo et al. found in 2020 that the Arabidopsis cytoplasmic ribosomal protein L14B was required for Arabidopsis fertilization (Luo et al., 2020). Therefore, it was speculated that the *Glyma.13G126400* gene might perform important functions during the process of ovule differentiation, thereby affecting the differentiation of seed pods in soybean. The *Glyma.13G126700* gene expresses the homology domain of Gnk2. Gnk2 is a small-molecule antibacterial peptide (Sawano et al., 2007). Its role in the formation of four-seed pods and ovule differentiation needs further research and verification. qRT-PCR results showed that both *Glyma.13G126400* and *Glyma.13G126700* might be positive regulators of PoFSP in soybean.

**Figure 11:**
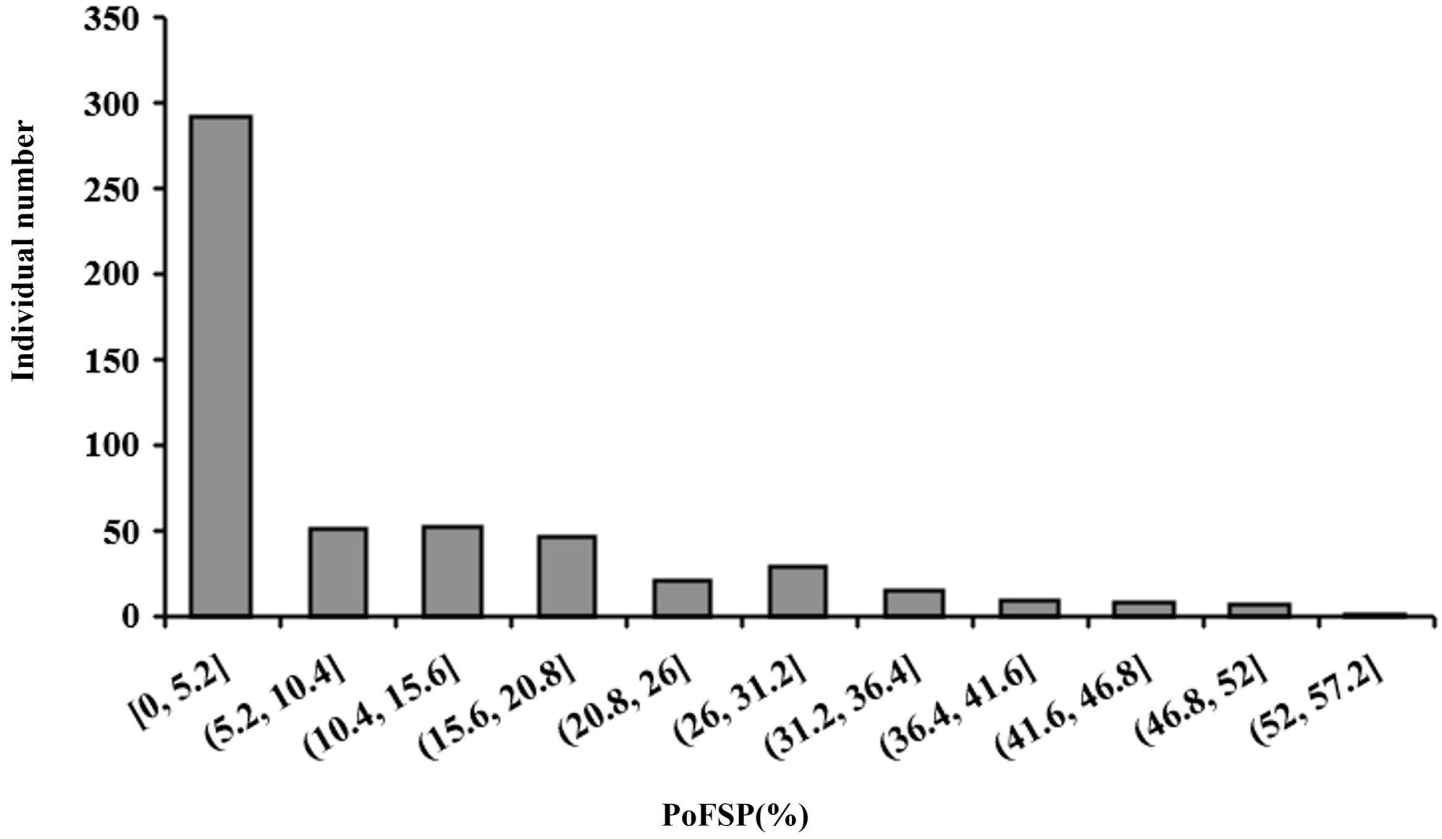
Frequency histogram of the phenotypic distribution of PoFSP in the resource population.

Haplotype analysis is an effective method to study the genetic diversity of genes using soybean germplasm resources. In this study, the resource population contained 527 varieties were used for haplotype analysis of the above five candidate genes for PoFSP. In 2021, the number of one, two, three and four-seed pods at the maturity stage of the resource population were investigated in the field, and PoFSP was calculated. In 2021, the maximum PoFSP in the resource population was 52.78%, the minimum value was 0, and the average value was 9.5%. Skewness and kurtosis were 1.4 and 1.2, respectively, and CV was 1.27 (**Table 4**; **Figure 11**). In more than half of the resource population contained 527 varieties, PoFSP was between 0 and 5%. Haplotype analysis of candidate genes for PoFSP in the resource population was helpful for the discovery of rare variation materials in PoFSP. The five candidate genes all had two or more major haplotypes in the resource population. Through significance analysis of phenotypic differences between major haplotypes, it was found that the five candidate genes all had haplotype differences in the resource population. In this study, the number of cultivars exceeded 5% of the total resource population to classify the major haplotypes. Although haplotypes with extreme phenotypes might be missed, major haplotypes could be mined more precisely for allelic diversity. According to the present results, the effects of the above five genes on soybean PoFSP were possible, but the specific effects and mechanisms of each gene on PoFSP need further study.

**Table 4:** Statistics analysis of PoFSP in the resource population.

Phenotype	Min	Mix	Mean	Standard Deviation	CV	Skewness	Kurtosis
PoFSP	0	52.78%	9.50%	12.11	1.27	1.40	1.20

## Conclusion

In this study, PoFSP was comapped to a QTL(*qPoFSP13-1*) on chromosome 13 in CSSLs by ICIM and BSA methods. F_2_ and RHL fine mapping populations were constructed for this QTL. The QTL was fine mapped between 23.91 Mb and 24.01 Mb on chromosome 13, and the interval size was 100 Kb. A total of 14 candidate genes were included in this QTL. Through sequence analysis, expression analysis and haplotype analysis, 5 candidate genes and their effects on PoFSP were analyzed and predicted in soybean.

## Data availability statement

The original contributions presented in the study are included in the article/**Supplementary Materials**. Further inquiries can be directed to the corresponding authors.

## Author contributions

ChL, HJ, and QC conceived the study and designed and managed the experiments. MY,XW and YZ, provided plant lines. FC, LH, CK, TZ and CS performed trials and collected data. FC, CaL and NW completed statistical analyses of phenotypic data. FC, RW and JX contributed to writing the paper. All authors contributed to the article and approved the submitted version.
