# Supplementary material for: Fine mapping and candidate gene analysis of proportion of four-seed pods by soybean CSSLs
Source: Front Plant Sci. 2023 Jan 18;13:1104022. doi: 10.3389/fpls.2022.1104022 (PMC9890659; doi:10.3389/fpls.2022.1104022)
Supplement: Supplementary file 2 [file Table_1.docx]

Supplementary Table 1 Proportion of four-seed pods QTLs found in CSSLs

| Years | Name | Chromosome | LOD | PVE% | ADD | Start (bp) | End (bp) |
| --- | --- | --- | --- | --- | --- | --- | --- |
| 2016 | *qPoFSP2-1* | Chr02 | 2.8912 | 4.4342 | -0.0157 | 3806257 | 3963691 |
| 2016 | *qPoFSP8-1* | Chr08 | 3.26 | 4.9468 | 0.0295 | 22668813 | 22841427 |
| 2016 | *qPoFSP16-1* | Chr16 | 4.8628 | 7.654 | 0.0347 | 31822329 | 31845684 |
| 2016 | *qPoFSP20-1* | Chr20 | 6.5822 | 11.3632 | -0.0582 | 35119142 | 35194650 |
| 2017 | *qPoFSP4-1* | Chr04 | 9.7045 | 12.168 | 0.0347 | 8807067 | 8829123 |
| 2017 | *qPoFSP4-2* | Chr04 | 2.9431 | 3.3815 | 0.0239 | 49678043 | 49904791 |
| 2017 | *qPoFSP9-1* | Chr09 | 7.3601 | 8.9018 | 0.0414 | 2437115 | 3511390 |
| 2017 | *qPoFSP10-1* | Chr10 | 2.7506 | 3.2461 | -0.0267 | 48132249 | 48264381 |
| 2018 | *qPoFSP4-3* | Chr04 | 4.7435 | 7.6585 | 0.0304 | 18201039 | 18426280 |
| 2018 | *qPoFSP12-1* | Chr12 | 2.5956 | 4.0247 | -0.0221 | 6792806 | 6880354 |
| BLUE | *qPoFSP8-2* | Chr08 | 5.6504 | 3.8829 | 0.0104 | 17536149 | 17560909 |
| BLUE | *qPoFSP16-2* | Chr16 | 3.369 | 2.2261 | 0.0139 | 31272178 | 31322499 |
| 2017 BLUE | *qPoFSP7-1* | Chr07 | 3.46 | 3.87 | 0.03 | 3900022 | 3934584 |
| 2017 BLUE | *qPoFSP7-2* | Chr07 | 10.5 | 7.44 | 0.02 | 39710561 | 39776712 |
| 2018 BLUE | *qPoFSP13-1* | Chr13 | 12.63 | 9.18 | 0.02 | 23976621 | 24036668 |
| 2018 BLUE | *qPoFSP17-1* | Chr17 | 4.65 | 3.05 | -0.02 | 2678066 | 2858489 |
| 2017 2018 BLUE | *qPoFSP20-2* | Chr20 | 33.31 | 30.69 | -0.05 | 35907647 | 35932855 |

Note: the “+” additive indicates that the additive effect comes from the allele of the wild parent ZYD00006.

Supplementary Table 2 Mapping QTL of the proportion of four-seed pods by BSA

| Chromosome | Distance (Mb) | Position | Gene Number |
| --- | --- | --- | --- |
| Chr01 | 0.09 | 120000 bp-210000 bp | 13 |
| Chr03 | 3.67 | 3010000 bp-6680000 bp | 309 |
| Chr08 | 0.10 | 24190000 bp-24290000 bp | 2 |
| Chr12 | 2.57 | 4800000 bp-7370000 bp | 413 |
| Chr13 | 3.61 | 22020000 bp-25630000 bp | 704 |
| Chr18 | 27.4 | 9770000 bp-37170000 bp | 924 |

Supplementary Table 3 Candidate gene annotation information

| Gene name | Position(bp) | Length(bp) | Arabidopsis homologous gene | Gene function prediction | Amino acid changes? |
| --- | --- | --- | --- | --- | --- |
| *Glyma.13G125800* | 23913893-23919430 | 5537 | AT5G49830 | Cullin repeat-like-containing domain | YES |
| *Glyma.13G125900* | 23921172-23923832 | 2660 | AT1G04030.1 |  | YES |
| *Glyma.13G126000* | 23922919-23927833 | 4914 | AT5G44030.2 |  | YES |
| *Glyma.13G126100* | 23945122-23945287 | 165 |  |  | YES |
| *Glyma.13G126200* | 23951476-23958210 | 6734 | AT3G04030.3 | MYB-CC type transfactor, LHEQLE motif | YES |
| *Glyma.13G126300* | 23969963-23971099 | 1136 |  |  | NO |
| *Glyma.13G126400* | 23974969-23976380 | 1411 | AT1G04480.1 | Ribosomal protein L14 domain | NO |
| *Glyma.13G126500* | 23978121-23979209 | 1088 | AT5G44000.1 | Glutathione S-transferase | YES |
| *Glyma.13G126600* | 23979723-23986655 | 6932 | AT1G04050.1 | Histone-lysine N-methyltransferase SUVR1/2/4 | YES |
| *Glyma.13G126700* | 23992460-23994040 | 1580 | AT5G43980.1 | Gnk2-homologous domain | NO |
| *Glyma.13G126800* | 23999716-23999869 | 153 |  |  | NO |
| *Glyma.13G126900* | 24000388-24001338 | 950 | AT1G04070.1 | Mitochondrial import receptor subunit Tom22 | NO |
| *Glyma.13G127000* | 24008532-24012615 | 4083 | AT1G04550.2 | AUX/IAA protein | NO |
| *Glyma.13G127100* | 24013026-24016923 | 3897 |  | Protein kinase domain | YES |

Supplementary Table 4 SNP mutations in the CDS region of candidate genes

| Gene name | Chr. | Position | Codon change | Amino acid change |
| --- | --- | --- | --- | --- |
| *Glyma.13G125800* | 13 | 23915183 | gaG/gaT | Glu/Asp |
|  |  | 23916402 | aAa/aGa | Lys/Arg |
|  |  | 23916733 | caT/caA | His/Gln |
|  |  | 23918923 | aaC/aaT | -- |
|  |  | 23918973 | gCt/gTt | Ala/Val |
| *Glyma.13G125900* | 13 | 23921909 | gaT/gaG | Asp/Glu |
|  |  | 23922112 | cAg/cTg | Gln/Leu |
|  |  | 23922738 | acC/acA | -- |
| *Glyma.13G126000* | 13 | 23924325 | ccA/ccG | -- |
|  |  | 23926406 | gCt/gTt | Ala/Val |
| *Glyma.13G126100* | 13 | 23945144 | gcC/gcT | -- |
|  |  | 23945266 | cCg/cAg | Pro/Gln |
| *Glyma.13G126200* | 13 | 23953513 | cTt/cCt | Leu/Pro |
| *Glyma.13G126500* | 13 | 23978916 | cCt/cGt | Pro/Arg |
|  |  | 23979190 | acA/acT | -- |
| *Glyma.13G126600* | 13 | 23980962 | Ccc/Gcc | Pro/Ala |
|  |  | 23981467 | Cca/Tca | Pro/Ser |
|  |  | 23982764 | atG/atA | Met/Ile |
|  |  | 23982984 | Gta/Ata | Val/Ile |
| *Glyma.13G127100* | 13 | 24014033 | Agg/Ggg | Arg/Gly |
|  |  | 24014267 | Gtg/Atg | Val/Met |
|  |  | 24014939 | Cga/Tga | Arg/end |

Supplementary Table 5 InDel mutation in the CDS region of candidate genes

| Gene name | Position | SN14 | ZYD00006 | Effects | Amino acid change |
| --- | --- | --- | --- | --- | --- |
| *Glyma.13G125800* | 23916567 | TCAC | T | CODON DELETION | AspHis/Asp |
| *Glyma.13G125900* | 23922739 | C | ACCC | CODON INSERTION | Ser/SerThr |

Supplementary Table 6 InDel mutation of the candidate gene promoter

| Gene name | Position | SN14 | ZYD00006 |
| --- | --- | --- | --- |
| *Glyma.13G125800* | 23911407 | TTT | T |
|  | 23912506 | A | AGACACGGACA |
| *Glyma.13G125900* | 23918201 | T | TT |
| *Glyma.13G126000* | 23928014 | C | CAAC |
| *Glyma.13G126100* | 23946312 | A | AA |
|  | 23946008 | T | TTTGT…TGTTG |
| *Glyma.13G126200* | 23960975 | GAAAG…GAGAT | T |
|  | 23960912 | G | GG |
|  | 23958330 | T | TT |
| *Glyma.13G126300* | 23974058 | T | TT |
| *Glyma.13G126400* | 23978597 | TT | T |
| *Glyma.13G126500* | 23977603 | C | CCCTT…TCATA |
| *Glyma.13G126600* | 23988708 | C | GAAAA…AGTTC |
|  | 23988608 | AA | A |
|  | 23987719 | T | TA |
| *Glyma.13G126700* | 23990742 | A | ATA |
| *Glyma.13G126800* | 23998096 | A | AA |
|  | 23998977 | A | ATTGAAAACTA |
|  | 23999060 | A | ACAA |
|  | 23999099 | A | AA |
| *Glyma.13G126900* | 23998153 | A | AA |
|  | 23999034 | A | ATTGAAAACTA |
|  | 23999117 | A | ACAA |
|  | 23999155 | A | AA |
|  | 23999886 | C | CCCAA…GGAGT |
| *Glyma.13G127000* | 24005538 | T | TTTCCT |
